# Electrophysiological changes in optic neuropathy of streptozotocin induced diabetic rats

**Published:** 2013-09-25

**Authors:** AM Ghita, D Parvu, R Sava, L Georgescu, L Zagrean

**Affiliations:** * University Emergency Hospital Bucharest, Romania; ** "Carol Davila" University of Medicine and Pharmacy, Bucharest, Romania

**Keywords:** visually evoked potential, blood glucose, glycemic values, diabetic neuropathy, diabetes mellitus

## Abstract

The visually evoked potentials are electrical signals generated by the occipital cortex due to electrical stimulus. The clinical importance of VEP is to diagnose the functional changes of the optic nerve in different diseases such as diabetic mellitus. Our study sought latency of VEP changes depending on glycemic value and duration of diabetes in Wistar rats.

** Methods: **this study evaluated the VEP of 25 rats in three groups: control group, diabetic group 1 with glycemic values between 200-400mg/dl and diabetic group 2 with glycemic values between 400 and 600mg/dl. These rats from diabetic group 2 were followed for 4 months and the ones in control group and diabetic group 1 for 5 months.

** Results:** the latency of VEP shows slight changes without any statistical significance in the control group. In diabetic group 1 and 2 similar changes occurred, with statistical significance and the amplitude of the changes was proportional with the glycemic value. The rats had a rapid increase of VEP latency after the induction of diabetes and returned to a normal range in the first month. After a time, when the latencies of VEP were in normal range, a new growth appeared faster and larger as the glycemic values were higher.

** Conclusion: **diabetes brings changes to the visual signal transmission and to the central processing, this being revealed by the examination of the visually evoked potential. Increased VEP latency is statistically correlated with the changes that occur at the level of the values of glucose in blood. A rapid growth in blood sugar lowers the visual signal transmission. This change is temporary despite the persistence of elevated blood glucose values, probably by adjusting to the new condition. However, maintaining high glycemic values remotely produces a progressive increase of the delay of the visual signal. This progressive increase is faster as blood glucose levels are higher.

## Introduction

Diabetic neuropathy, a common complication in patients with diabetes mellitus, can negatively influence the quality of life of affected individuals. The symptoms are various and are in strong connection with the illness length, glycemic values and other comorbidities. Diabetic neuropathy manifestations can be classified according to the location of the affected nerve in peripheral, autonomic nervous systems and central nervous system. While peripheral and autonomic nervous systems manifestations are widely studied, central nervous changes are less investigated. These changes can be proved by radiological, psychological and electrophysiological tests which show the alteration of answer to visual, auditory and somatosensory stimuli [**1,2**]. 

 Because of the extensive damage of the retina and the optic nerve, diabetic patients are difficult to diagnose and are rarely investigated for a possible association with the optic neuropathy. The nervous damage adds to the retinopathy and causes a loss of visual function and consequently a worsened prognosis [**3**]. Even though the changes in the optic nerve electrophysiology are more subtle and occur later, their repercussions on the visual function are severe. In humans, diabetes can result in diabetic papillopathy, anterior ischemic optic neuropathy, posterior ischemic optic neuropathy, papillary depigmentation (partial optic nerve atrophy) [**4**]. 

 Researches on the optic neuropathy are conducted on animals and usually investigate the peripheral modifications, both sensitive and motor pathways [**5-8**]. For studying the central nervous system modifications on diabetic animals, visual, motor and somatosensory evoked potentials can be used [**2,9**]. Histopathological tests show that the optic nerve is severely damaged because of the loss of nervous fibers [**9**]. 

 An important drawback of these studies of neuro-electrophysiological diabetes induced modifications comes from the lack of homogeneity of the way of inducing diabetes, the age at which diabetes is induced, the chosen laboratory animals and the selected tests and parameters. Most studies on animal models are performed under anesthesia, which induces difficulties in a correct analysis of the test parameters [**10**]. Using the same level of anesthetic depth, lowers the variability induced by anesthetic which is essential for a correct assessment of diabetic neuropathy.

## Materials and methods

25 male Wistar rats, aged approximately 3 months and weighing 300-350 g were followed for the experiment.

**The induction of diabetes and the dividing in groups**

 Rats were divided into three groups: control group (non-diabetic rats), group 1 rats with diabetes with moderately elevated values of blood glucose (between 200 and 400mg/dl) and group 2 - rats with diabetes with very high blood sugar (between 400 and 600mg). Induction of diabetes was achieved by an intraperitoneal injection of a dose of 50 mg/kg of streptozotocin in 0,9% NaCl. After the injection, different glucose values between normal and very high (over 600 mg / dl) were obtained. Rats with glucose values below 200 g/dl were removed from the study due to recurrence of normal glucose values in 1-4 weeks. Rats with values above 600mg were also eliminated from the study due to poor biological potential which would not ensure their survival for a minimum follow-up period of 16 weeks. 3 groups were formed: 10 rats in the control group, 7 rats in group 1 and 8 rats in group 2. Control group and group 1 were followed for 5 months and group 3 was followed for 4 months. After month 4, in the untreated rats in group 2, metabolic changes, weight loss and acquired infections have appeared and resulted in death of five of them. These experimental groups were recorded under anesthesia with chloral hydrate. The animals were kept in transparent plastic boxes with free access to food and water and ambient temperature of 23 degrees Celsius.

** Chronically implanted rats**

 Visually evoked potentials were recorded with a chronic implant performed under general anesthesia with intraperitoneal injection with chloral hydrate (0.4g/kgc). The implantation was performed two weeks before the induction of diabetes. The chronic implantation was performed after ensuring an efficient performance of the anesthesia. Head fixation was done by using a stereotaxic device for immobilization. Mounting electrodes was performed directly without using any adjacent substances. The material used for making the electrode was nickel-chromium Alloy (ni80cr20, diameter 0.15 mm). We used 3 electrodes, 2 of them active positioned 6 mm posteriorly and 4 mm laterally to the bregma (corresponding visual area 17) and a reference eletrode positioned 7 mm anteriorly to the bregma (corresponding olfactory bulb). In the first stage of implantation we made a median incision and afterwards we removed the adjacent tissues from the scalp. Later we performed 2 trepanation for each electrode, leaving between them 1 mm of bone bridge for subsequent anchoring the electrode.

** Electrode implantation stages in a Wistar rat.**

 VEP recordings were made after a predetermined schedule. Rats in the diabetic group were registered at the time of induction of diabetes, at 2 weeks and then monthly for 4 months for group 2 and for 5 months for group 1. Recordings were performed under anesthesia with chloral hydrate (0.4 g/kg) injected intraperitonealy and PEV were recorded sequentially from the induction to awakening. All recordings were performed in a dark room; soundproofing and visual potentials were evoked by applying light stimuli placed at 1 cm from the eye at an angle of 45 degrees superior to the horizontal plane of the eye. Contra lateral eye was covered with an opaque tape. Light flashes were generated at a frequency of 30 stimuli /minute with the duration of stimulus of 0.015s. In order to obtain VEPs, we averaged 300 responses to the stimuli taken in a period of 5 minutes.

 The electrodes were connected to Biopac MP150 system and the data were acquired on a computer with Acqknowledge 4.2 software and the impedance of the electrodes was under 100Mω. The signal was amplified 5000 times and artifacts and interferences were removed with a bandpass filter of 1-100Hz. Data were recorded with 1000 frames per second. Data analysis identified five peaks of visual evoked potentials where positive peaks were noted with P and negatives with N. We measured amplitudes and latencies for each one.

 Data analysis was conducted according to the classification in groups and the time from the induction of diabetes. To remove changes induced by the anesthetic, rats were registered at similar anesthetic depth (EEG median frequency> 4.5 Hz).

 Spectral analysis of electrical recording was performed with acquisition program Acqknowledge 4.2. Median frequency (FM) rate was calculated for a 5 minute recording by averaging the values calculated from age 4 seconds. Median frequency (FM) represents the frequency at which 50% of the total spectral power of an epoch is located.

## Results

For a good outline of latency variability, all rats were recorded in the superficial anesthesia state with a median EEG frequency greater than 4.5 Hz (MF>4.5 Hz. Analyzing of the VEP included measurements of all 5 wave latencies N1, N2, N3 and P1, P2 but, reliable peacks proved to be only N1, P1 and N2. Evoked potential morphology changes during anesthesia along with loss of depth such as variability of P2, N3 which is very high, thus making it difficult to interpret and use in statistical processing.

**Fig. 1:**
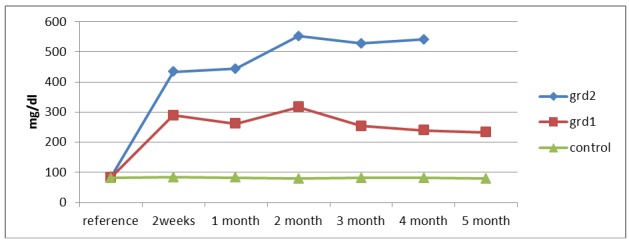
Average glycemic control group, group 1 (200-400 mg) and group 2 (400-600 mg).

 Glycemic values of rats were measured at each recording with a glucose meter. We mediated blood glucose for each group and for each registration period. Note that in Figure 1 the mean blood glucose values for the three groups are similar, with no statistical differences at the time of induction of diabetes. After induction, rats were included in one of two groups: group 1 with moderately elevated values (200-400 mg / dl) and group 2 with very high (400-600 mg / dl). The values were statistically different from the control group and between the two groups with diabetes.

 Examining the latency of the control group (no diabetes induced) we observed a slight variation. There was no statistically significant difference between the recordings for both their latency and amplitude.

 N1 peak latency analysis of visual evoked potentials shows different dynamics for each group of Wistar rats. Variability is minimal in the control group during the 5 months of examination. A slight increase in latency occurs at 2 weeks after induction of diabetes in diabetic group 1 (glucose 200-400mg/dl) and returned to baseline after 1 month after induction of diabetes. Latency values are maintained in the normal range until the month 5 when there is an increase in latency. For group 2 (glucose 400 -600mg/dl), there is a rapid and more extensive increase of the latency N1 than in group 1, with a return to normal in a month. N1 latency then begins to increase progressively from the third month.

**Fig. 2:**
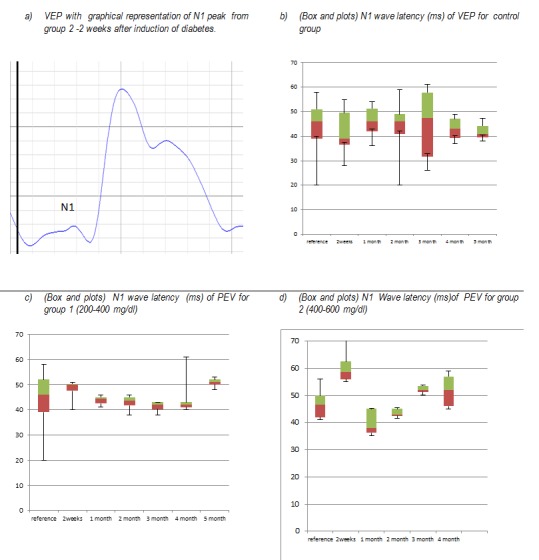
N1 wave of the VEP- morphology and latency in the three groups examined

 P1 latency shows dynamics similar to the dynamics of each group where we examined N1 latency. 

 Latency values in the non-diabetic group are relatively constant in successive examinations performed. In group 1, there is a slight increase without statistical difference (p =)? compared with baseline latency of P1 and then we observe a rapid return to normal levels of latency. Group 2 shows the highest variability with a rapid increase of latency at 2 weeks, but later it manifests a much slower recovery at two months. Then we observe a progressive increase starting the third month.

**Fig. 3:**
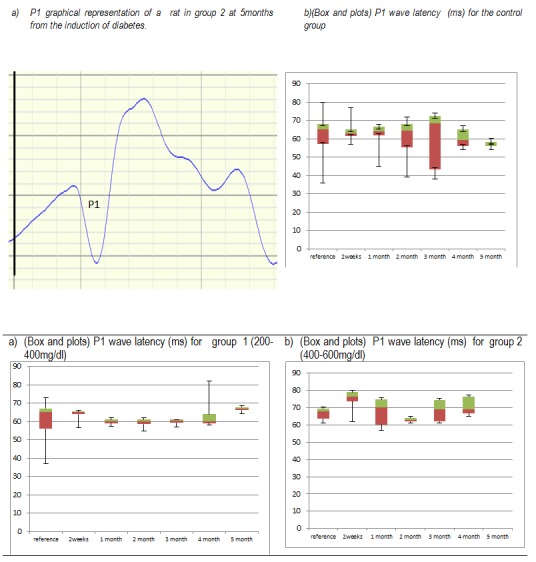
P1 wave of VEP - morphology and latency in the three groups examined

 N2 peak latency changes are similar to the peaks P1 and N1. Control group does not show significant variability of latency. In group 1 there is a slight increase in average latency N2 at 2 weeks after induction of diabetes but without statistical difference as far as the initial values are regarded and a slower recovery compared to N1 and P1 waves, going step-by-step to normal values. Three months after implantation, the mean of N2 latency reaches a minimum in group 1, then, starting with the fourth month there is a progressive increase. In group 2, the average N2 latency is similar to the one from group 1, but the modification is statistically significant compared to the initial values. After reaching the minimum values in the second month after the induction of diabetes, starting with the third month, the medium of N2 latency increases progressively. 

**Fig. 4:**
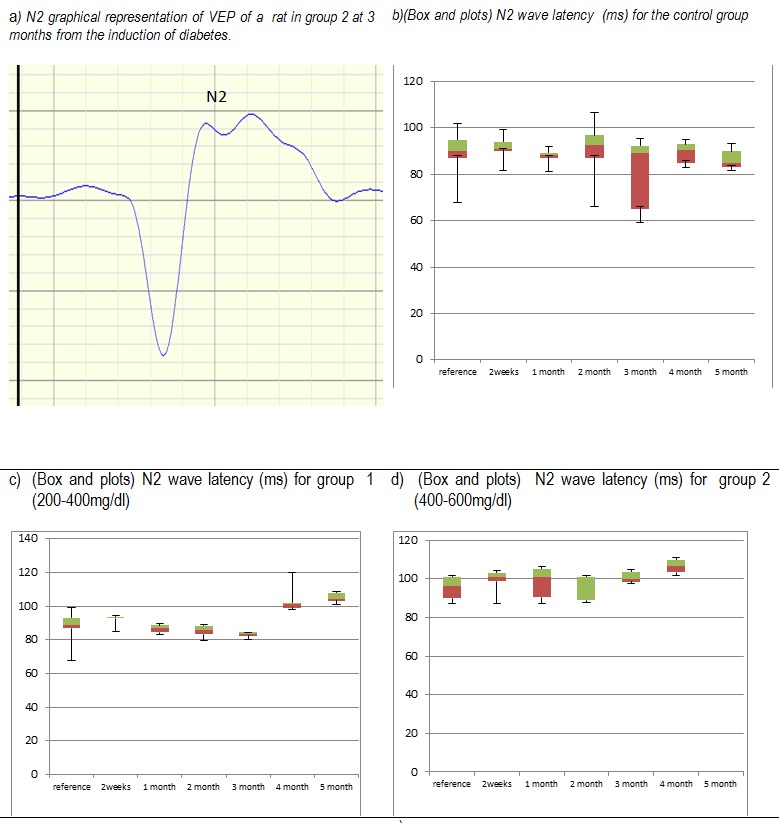
Fig. 4

 N2 and P2 latencies and morphology show large variability even in the same rat successive recordings and the same level of depth. However, shows a consistent average latency for the first three peaks that N1, P1, N2. The results for each group are presented in Table 1.

**Table 1:** Average of N2 and P3 latencies in rats from control group and groups 1 and 2.

	Control gr.		Gr 1 -diabetes (200-400mg/dl)		Gr 2- diabetes (200-400mg/dl)	
time	P2 (ms)	dev	P2 (ms)	dev	P2 (ms)	dev
reference	116,7167	19,07265	114,875	18,97885	129	9,899495
2 weeks	123,8696	13,47491	122,75	2,217356	126,5	18,04792
1 month	119,6667	18,18258	136,8333	21,2077	135,7778	18,61973
2 mouth	124,8125	17,11615	122,875	15,48675	139,7667	7,709734
3 mouth	104,75	21,41261	118,5	7,937254	129,3333	7,033254
4 mouth	122,8	18,7664	123,8	6,978539	137,25	15,80009
5 mouth	113,5455	9,384707	130,3333	4,226898		
	N3 (ms)	dev	N3	dev	N3	dev
reference	135,2833	21,41051	132,625	19,71998	151	17,24038
2 weeks	145,6957	18,26182	137	4,690416	151,1667	31,39436
1 mouth	144,1667	25,27245	157,3333	21,96057	157,5556	25,26911
2 mouth	140,75	18,08683	134,5	16,84382	168,1	9,174148
3 mouth	139,25	39,43077	132	8,445906	144,3333	4,273952
4 mouth	134,3	20,1166	138,8	13,10343	163,125	10,58891
5 mouth	121,3273	4,995616	144,6667	7,788881		

 Comparing the results for the control group, group 1 and 2 diabetes, we observed different dynamics of latency. For groups of diabetes there is an increase in the mean N1 peak latency after the induction of diabetes with statistically different values compared to the ones in the control group, and then a return to normal. Starting with the third month N1 latency average for Group 2 increases progressively (statistically different (p <0.01) from the control group). For group 1 we observe a noticeable increase in N1 latency from the 4th month, but it is significantly higher than the control group only from the 5th follow-up month (p <0.01). 

**Fig. 5:**
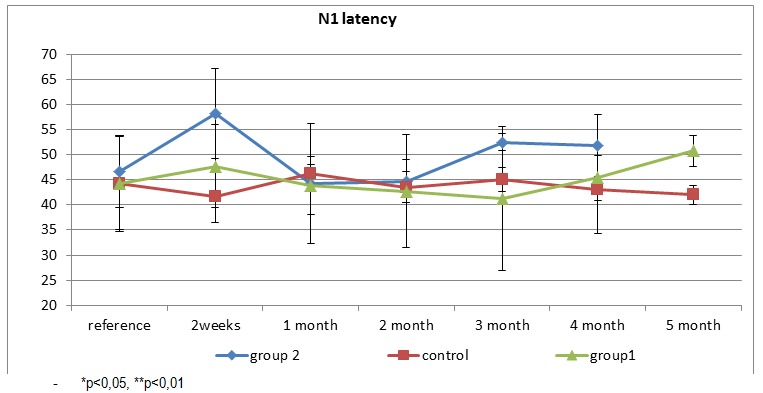
Average N1 latency for each group (control, diabetic 1 and 2)

 Examining the P1 peak latency in both diabetic groups, we observed the same rapid increase of latency after induction of diabetes. In group 2, the change is statistically significant compared to the control group. After the peak value in week 2, P1 values return to the ones similar to the control group, maintaining over them, but without any statistical differences. Starting with the third month, latency average of P1 gradually increases (with statistical significance compared to the control group (p <0.01)). In group 1, P1 latency values are slightly elevated compared to controls but there is no statistical difference between the two groups in the first four months of follow up. In the fifth month, P1 latencies grow and are statistically different from the control group (p <0.05).

**Fig. 6:**
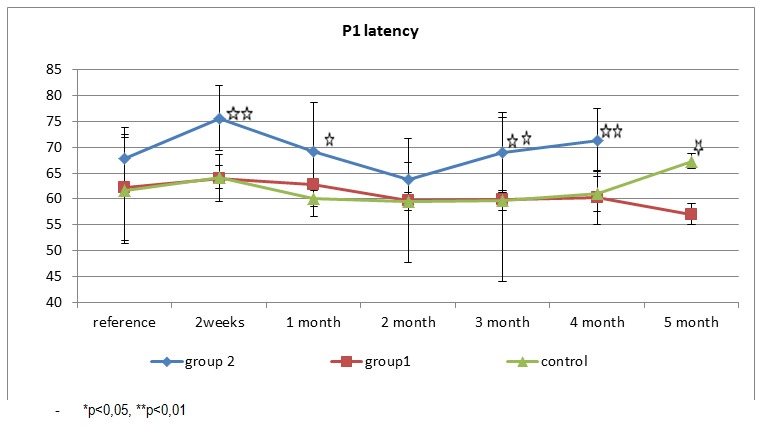
Average P1 latency for each group (control, diabetic 1 and 2)

 For N2 peak latency we observed the same behavior and statistical significance with P1 latency. Increased N2 latency in group 2 occurs from the 4th month of tracking and the increase is statistically significant compared with the control group.

**Fig. 7:**
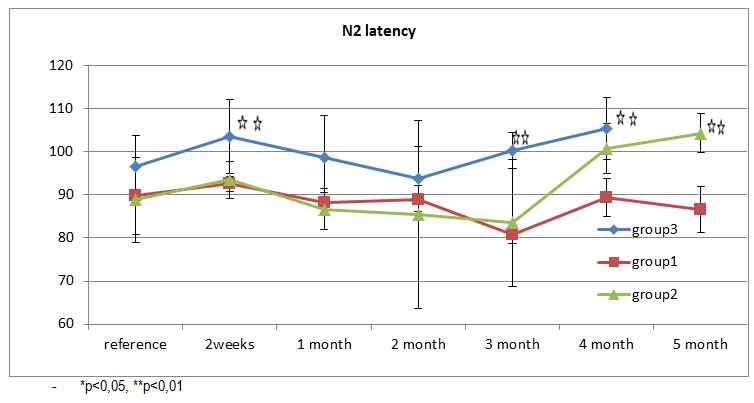
Average N2 latency for each group (control, diabetic 1 and 2)

## Conclusion

The results we obtained in Wistar rats with streptozotocin-induced diabetes are in concordance with previous data in literature. In order to reduce the variability of VEPs, we recorded VEPs at the same anesthetic depth. In diabetic rats, the latencies of peaks N1, P1, N2 and P2 increase at two weeks from diabetes induction in comparison to the baseline recording and to the control group. The increase in latency is statistically significant for peak N1 in group 1 (p<0,05) and for all peaks in group 2 (p<0,001). The average latency is higher and more dispersed in group 2 (N1 58,17ms+/-8,99 si N2 103,58+/-8,68) compared to group 1 (N1 47,75+/-5,18 si N2 93,5+/-1,73). Towards one month after diabetes induction, latency values decline towards baseline values.

 In both group 1 and 2, VEP latencies recorded starting with one-month post-induction of diabetes are near baseline values without any significant differences between the two groups. In group 2, the latencies of the VEP remain near-baseline until the fourth month post-induction. Starting with month four there is an important increase in the average of VEP latencies. Differences in the average latencies between the control group (N1 41,98 ms+/-3,09, N2 86,61ms+/-4,58) and group 1 (N1 50,83ms+/-1,83, N2 104,34ms+/-5,35) are statistically significant for all peaks (p<0,001) starting with the fifth month post-induction.

 After the pseudonormalization of VEP values starting with month one, group 2 maintains near-baseline values for a shorter duration than group one, one month). Even if the average of latencies is near-baseline and there are no statistically significant differences for most VEP with the exception of peak N1, they are still above the average latencies of the control group. Starting with the third month post-induction, there is a progressive increase in VEP latencies in group 3 (N1 52,5ms+/-1,64, N2 100,33ms +/-4,22) that is statistically significant from the control group (N1 45ms+/-14,36 and N3 80,87ms +/-14,72) (p<0,001). This increase in latencies can be attributed to the worsening of diabetic lesions occurring in both groups and more intensely in the higher-glycemic level group 2.

## Discussion

 For a good comparison of the results we used Wistar rats with similar initial weight (330 grams), kept in similar conditions and recorded under the same anesthetic depth. There are some limitations of the study. A first limitation is the inability to obtain viable rats with blood glucose over 400 mg / dl for a period exceeding four months. This is due to significant health alterations such as weight loss, infections and metabolic changes that can occur. A second limitation of the study is the absence of a histological examination of the optic nerve that could correlate functional changes with histological changes.

 An important variation of the diabetes-induced latencies of all PEV peaks (statistically significant) has been observed compared with the control group. Dynamic of the changes is similar in both groups of diabetes with a rapid growth of VEP latencies at 2 weeks after induction and then a return for a short period and again an increase.

 Initial changes seem to be related to an optic nerve dysfunction induced by sharply increased glucose values [**7,11,12**] and less to structurally changed axons. This dysfunction is related to the glycemic values. The restitution to normal values shows an adaptation of the nerve to high blood sugar. However, high glycemic values in group 2 appear to undergo a partial adaptation because VEP latencies values, except N1 latency, are always above the values of the control group, even though there is no statistical significance. In the second period of tracking diabetic rats, the latency increased again faster and ampler in the group with high blood sugar compared with the group with moderately increased blood glucose. In this stage, according to previous studies [**9,13,14**], it occurs a structural change of the optic nerve. We assume that histological changes occur faster and are more significant the greater blood glucose value is.

 To conclude, diabetes brings changes to the visual signal transmission and to the central processing, this being revealed by the examination of the visual evoked potential. Increased VEP latency is statistically correlated with the changes in blood glucose values. A rapid growth in blood sugar lowers the visual signal transmission. This change is temporary despite the persistence of elevated blood glucose values, probably by adjusting to the new condition. However, maintaining high glycemic values remotely produces a progressive increase of the delay of the visual signal. This progressive increase is faster as blood glucose levels are higher.
